# Harnessing Metformin’s Immunomodulatory Effects on Immune Cells to Combat Breast Cancer

**DOI:** 10.3390/ijms25115869

**Published:** 2024-05-28

**Authors:** Andjela Petrovic, Ivan Jovanovic, Bojan Stojanovic, Milica Dimitrijevic Stojanovic, Bojana S. Stojanovic, Milena Jurisevic, Bojana Simovic Markovic, Marina Jovanovic, Milan Jovanovic, Mihailo Jovanovic, Nevena Gajovic

**Affiliations:** 1 Center for Molecular Medicine and Stem Cell Research, Faculty of Medical Sciences, University of Kragujevac, Svetozara Markovica 69, 34000 Kragujevac, Serbia; petrovicandjela9944@gmail.com (A.P.); ivanjovanovic77@gmail.com (I.J.); milicadimitrijevic@yahoo.com (M.D.S.); bojanstojanovic@fmn.kg.ac.rs (B.S.S.); bojana.simovic@gmail.com (B.S.M.); marina_jovanovic@rocketmail.com (M.J.); gajovicnevena@yahoo.com (N.G.); 2 Department of Surgery, Faculty of Medical Sciences, University of Kragujevac, Svetozara Markovica 69, 34000 Kragujevac, Serbia; 3 Department of Pathology, Faculty of Medical Sciences, University of Kragujevac, Svetozara Markovica 69, 34000 Kragujevac, Serbia; 4 Department of Pathophysiology, Faculty of Medical Sciences, University of Kragujevac, Svetozara Markovica 69, 34000 Kragujevac, Serbia; 5 Department of Pharmacy, Faculty of Medical Sciences, University of Kragujevac, Svetozara Markovica 69, 34000 Kragujevac, Serbia; milena.jurisevic13@gmail.com; 6 Department of Otorhinolaryngology, Faculty of Medical Sciences, Svetozara Markovica 69, 34000 Kragujevac, Serbia; 7 Department of Abdominal Surgery, Military Medical Academy, University of Defence, Crnotravska 17, 11000 Belgrade, Serbia; milan.jovanovic@vma.mod.gov.rs; 8 Clinic for Orthopaedics and Traumatology, University Clinical Center, Zmaj Jovina 30, 34000 Kragujevac, Serbia; dok992@gmail.com

**Keywords:** metformin, breast cancer, NKT cells, Tregs, MDSCs, STAT4, NFAT

## Abstract

Metformin, a medication known for its anti-glycemic properties, also demonstrates potent immune system activation. In our study, using a 4T1 breast cancer model in BALB/C WT mice, we examined metformin’s impact on the functional phenotype of multiple immune cells, with a specific emphasis on natural killer T (NKT) cells due to their understudied role in this context. Metformin administration delayed the appearance and growth of carcinoma. Furthermore, metformin increased the percentage of IFN-γ^+^ NKT cells, and enhanced CD107a expression, as measured by MFI, while decreasing PD-1^+^, FoxP3^+^, and IL-10^+^ NKT cells in spleens of metformin-treated mice. In primary tumors, metformin increased the percentage of NKp46^+^ NKT cells and increased FasL expression, while lowering the percentages of FoxP3^+^, PD-1^+^, and IL-10-producing NKT cells and KLRG1 expression. Activation markers increased, and immunosuppressive markers declined in T cells from both the spleen and tumors. Furthermore, metformin decreased IL-10^+^ and FoxP3^+^ Tregs, along with Gr-1^+^ myeloid-derived suppressor cells (MDSCs) in spleens, and in tumor tissue, it decreased IL-10^+^ and FoxP3^+^ Tregs, Gr-1^+^, NF-κB^+^, and iNOS^+^ MDSCs, and iNOS^+^ dendritic cells (DCs), while increasing the DCs quantity. Additionally, increased expression levels of MIP1a, STAT4, and NFAT in splenocytes were found. These comprehensive findings illustrate metformin’s broad immunomodulatory impact across a variety of immune cells, including stimulating NKT cells and T cells, while inhibiting Tregs and MDSCs. This dynamic modulation may potentiate its use in cancer immunotherapy, highlighting its potential to modulate the tumor microenvironment across a spectrum of immune cell types.

## 1. Introduction

As of the year 2020, breast cancer stands as the most commonly diagnosed neoplasia in women worldwide, with an annual incidence of over 2 million new cases. This represents a remarkable 25.8% of all cancer cases in women. Despite substantial advancements in cancer treatment, recent data indicate that this malignancy remains at the top of the fatality rate scale [1]. The primary cause of such a low survival rate is the tumor’s exceptional capacity to develop distant metastasis, particularly in the lungs, bones, brain, liver, and lymphatic nodes [2].

Metformin is an oral anti-glycemic drug that has demonstrated remarkable efficacy in regulating blood glucose levels but is also used for polycystic ovaries, obesity, and cardiovascular conditions [3]. In the past few decades, metformin has emerged as a potent activator of the immune system and a promising ally in the fight against tumors [4]. The impact of metformin on cells of both the innate and the acquired immune response and its potential in combating tumors has been the focus of numerous years of investigation [5]. Natural killer T (NKT) cells are a unique subset of immune cells that exhibit distinct characteristics that differentiate them from both T lymphocytes and natural killer (NK) cells. As part of both innate and specific immunity, they are characterized by numerous receptors and traits of NK cells as key representatives of innate immunity responses as well as T lymphocytes, as a crucial component of acquired immunity [6,7]. These specialized immune cells significantly contribute to the functioning of the immune system [8,9]. Considering the important role of NKT cells in immunoregulation and their diverse functions, our goal is to determine whether metformin affects this small yet powerful subset of cells and, if so, in what manner. To the best of our knowledge, this type of research has not been conducted to date.

## 2. Results

Our study was conducted using a 4T1 breast cancer model in BALB/C wild-type (WT) mice. Tumor induction was performed by subcutaneous injection of 5 × 10^4^ 4T1 cells diluted in 50 μL of the complete medium into the fourth mammary fat pad (MFP) of each mouse. The mice were divided into two groups: one treated with metformin at a dose of 200 mg/kg, diluted in 100 μL of distilled water, administered daily, and a non-treated group that received 100 μL of distilled water daily. The mice were humanely euthanized on the 35th day of the experiment and blood was taken for cytokine measurement as well as spleens and tumors for cell analyses.

### 2.1. Metformin Administration Results in Delayed Appearance of the Palpable Tumor and Slower Progression of 4T1 Breast Cancer

**Figure 1:**
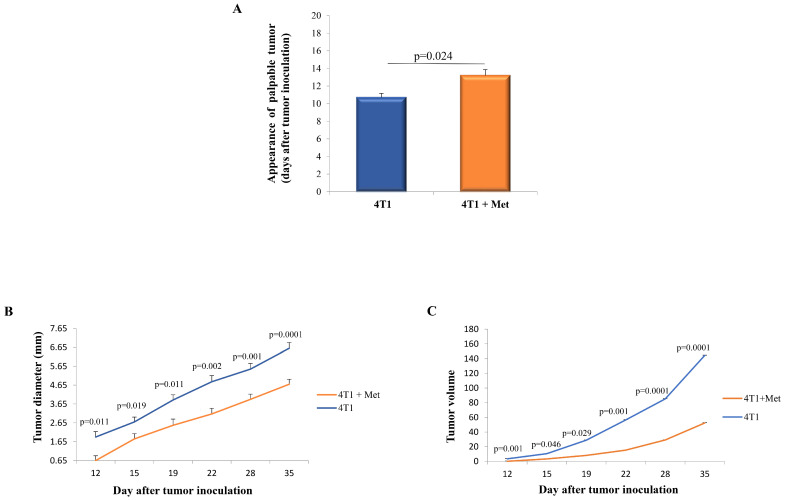
Metformin inhibits the appearance and growth of 4T1 breast carcinoma in WT mice. Mice were given 5 × 10^4^ 4T1 breast carcinoma cells. The experimental group of mice was subjected to daily metformin treatment, while the non-treated group was administered only distilled water. Palpable tumors were systematically monitored and measured at specific time points throughout the study period, specifically on days 12, 15, 19, 22, 28, and 35 (**A**,**B**). The tumor volume was quantitatively assessed on the same days (**C**). The data are shown as mean ± S.E.M. of six mice per group and are representative of three separate experiments. Statistical significance at each time point was calculated using the Student’s *t*-test.

The appearance of the palpable tumors occurred significantly faster in the group of non-treated mice compared to the group of metformin-treated mice (*p* = 0.024; Figure 1A). Furthermore, the diameter of the tumors was significantly smaller in the metformin-treated group, with the difference becoming statistically significant 12 days after the application of 4T1 tumor cells (*p* = 0.011; Figure 1B). Additionally, the tumor volume was significantly smaller in the metformin-treated group compared to the non-treated group (*p* = 0.001; Figure 1C).

### 2.2. Metformin Decreases Serum Levels of Interleukin (IL)-10

**Figure 2:**
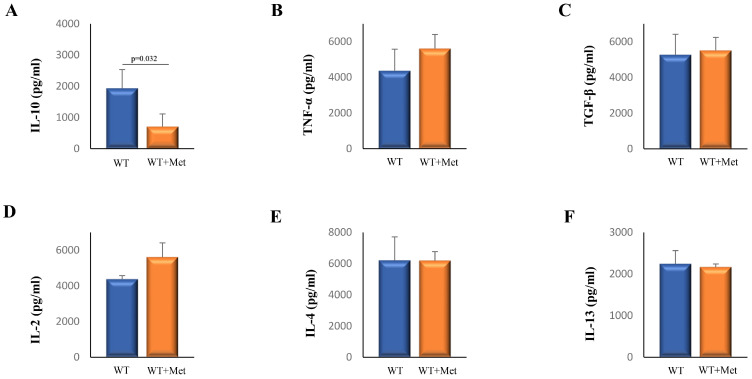
The effect of metformin on the cytokine profile in tumor-bearing mice. The levels of IL-10 (**A**), TNF-α (**B**), TGF-β (**C**), IL-2 (**D**), IL-4 (**E**), and IL-13 (**F**) derived from sera of metformin-treated and non-treated mice were quantified using the enzyme-linked immunosorbent assay (ELISA) method. Data are presented as mean ± S.E.M. for six mice per group, representative of three independent experiments. Statistical comparisons were made using the Student’s *t*-test.

In order to test the effect of metformin on the antitumor immune response, firstly we investigated the serum levels of pro- and anti-inflammatory cytokines. The analysis of serum cytokines revealed a decreased concentration of IL-10 in mice treated with metformin compared to the non-treated group (Figure 2A). Conversely, no significant differences were observed in the levels of tumor necrosis factor-alpha (TNFα), transforming growth factor-beta (TGF-β), IL-2, IL-4, and IL-13, between the mentioned groups (Figure 2B–F).

### 2.3. Metformin Treatment Suppresses the Inhibitory Phenotype of NKT Cells While Enhancing Their Cytotoxic Potential

**Figure 3:**
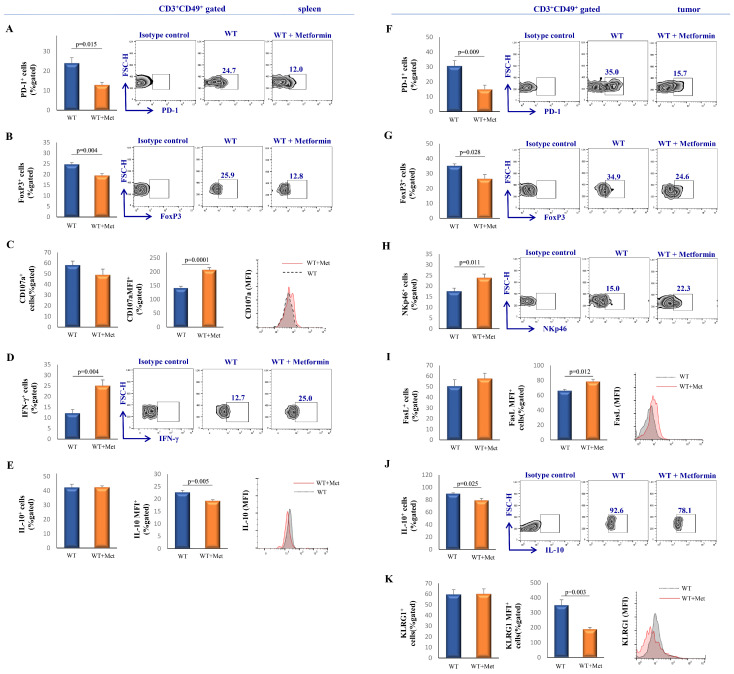
Metformin therapy alters the phenotype of CD3+CD49b+ cells toward tumorocidal type. The percentage of PD-1^+^ (**A**), FoxP3^+^ (**B**), CD107a MFI^+^ (**C**), IFN-γ^+^(**D**), and IL-10 MFI^+^ (**E**) CD3^+^CD49^+^ cells isolated from spleens and PD-1^+^ (**F**), FoxP3^+^ (**G**), NKp46^+^ (**H**), FasL MFI^+^ (**I**), IL-10^+^ (**J**) and KLRG1 MFI^+^ (**K**) CD3^+^CD49b^+^, derived from the primary tumor of metformin-treated and non-treated mice, was quantified and displayed on graphs and representative FACS plots. The data are shown as mean ± S.E.M. of six mice per group and are representative of three separate experiments. Statistical significance between groups was determined using the Student’s *t*-test.

In order to further demonstrate the potential immunomodulatory impact of metformin, we also examined the phenotype of splenic NKT cells. The data revealed a significantly lower percentage of programmed death-1 (PD-1)^+^ (*p* = 0.015) and forkhead box P3 (FoxP3)^+^ (*p* = 0.004) CD3^+^CD49^+^ cells in the metformin-treated group compared to the non-treated group (Figure 3A,B). Although there was no significant difference in the percentage of CD107a^+^ CD3^+^CD49^+^ cells between the groups, analysis of mean fluorescence intensity (MFI) showed significantly higher expression of CD107a (*p* = 0.0001) on individual CD3^+^CD49^+^ cells isolated from metformin-treated mice (Figure 3C). Intracellular staining revealed a significant increase in the percentage of interferon-gamma (IFN-γ)^+^ CD3^+^CD49^+^ cells (*p* = 0.004) as well as a lower expression of IL-10 per CD3^+^CD49^+^ cell (*p* = 0.005) in the metformin-treated group in comparison to the control group (Figure 2E and Figure 3D).

Phenotypic analyses of tumor-infiltrating cells revealed significantly lower percentages of PD-1^+^ (*p* = 0.009) and FoxP3^+^ (*p* = 0.028), and a higher percentage of NKp46^+^ (*p* = 0.011) CD3^+^CD49^+^ cells in the metformin-treated group (Figure 3F–H). A significant increase in MFI expression of the Fas ligand (FasL)^+^ (*p* = 0.012) and a decrease in the killer cell lectin-like receptor subfamily G member 1 (KLRG1)^+^ (*p* = 0.003) per CD3^+^CD49^+^ cell was observed in the metformin-treated group (Figure 3I,K). Moreover, we found a significantly decreased percentage of IL-10-producing CD3^+^CD49^+^ cells (*p* = 0.025) in the metformin-treated group of mice (Figure 3J).

### 2.4. Metformin Modulates the Functional Phenotype of Splenic and Tumor-Infiltrating T Cells in Tumor-Bearing Mice

**Figure 4:**
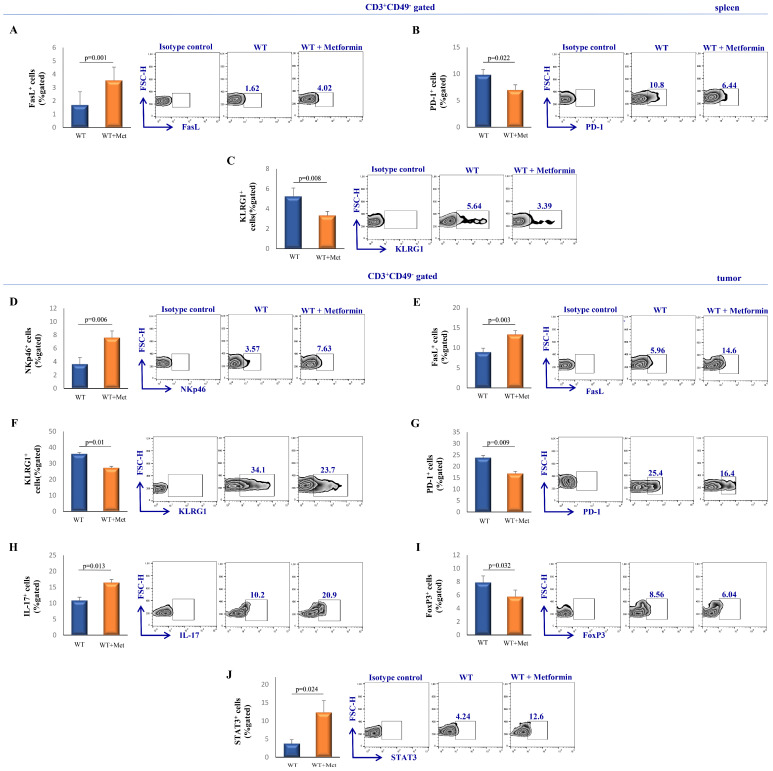
Metformin treatment modified the functional phenotype of splenic and tumor-infiltrating CD3^+^CD49^−^ cells in tumor-bearing mice. The percentage of FasL^+^ (**A**), PD-1^+^ (**B**), KLRG-1^+^ (**C**), in splenic CD3^+^CD49^−^ cells and NKp46^+^ (**D**), FasL^+^ (**E**), KLRG-1^+^ (**F**), PD-1^+^ (**G**), IL-17^+^ (**H**), FoxP3^+^ (**I**), STAT3^+^ (**J**) in tumor-infiltrating CD3^+^CD49^−^ cells derived from the primary tumor of metformin-treated and non-treated mice. Results were evaluated using flow cytometry and are presented on graphs and representative FACS plots as mean ± S.E.M. of six mice per group. A paired Student’s *t*-test was used to determine statistical significance.

In order to investigate the potential effects of metformin on other immune cells, we further investigated the phenotype of splenic and tumor-infiltrating T cells. A significantly elevated percentage of FasL^+^ (*p* = 0.001) and decreased percentage of PD-1^+^ (*p* = 0.022) and KLRG1^+^ (*p* = 0.008) CD3^+^CD49^−^ T cells were observed in spleens of metformin-treated mice compared to the control group (Figure 3B,C and Figure 4A).

Analysis of T cells in tumor tissue revealed a significantly increased percentage of NKp46^+^ (*p* = 0.006) and FasL^+^ (*p* = 0.003) and a decreased percentage of KLRG1^+^ (*p* = 0.01), and PD-1^+^ (*p* = 0.009) of CD3^+^CD49^−^ cells in the metformin-treated group compared to the control group (Figure 4D–G). Additionally, metformin increased the percentage of IL-17-producing CD3^+^CD49^−^ cells (*p* = 0.013, Figure 4H). Finally, a significant decrease in FoxP3 expression (*p* = 0.032) and an increase in signal transducer and activator of transcription 3 (STAT3) (*p* = 0.024) expression were measured in tumor-derived CD3^+^CD49^−^ cells isolated from the metformin-treated group of mice (Figure 4I,J).

### 2.5. Administration of Metformin Attenuates the Accumulation and Activation of Immunosuppressive Cell Populations

**Figure 5:**
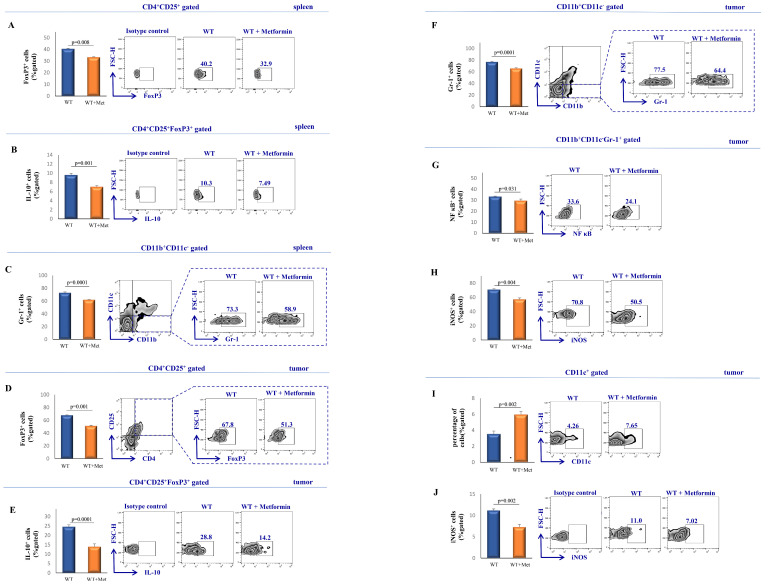
Metformin administration induces notable changes in Tregs, MDSCs, and dendritic cells isolated from spleens and tumors. The graphs and representative FACS plots illustrate the percentage of splenic FoxP3^+^CD4^+^CD25^+^ (**A**), IL-10^+^CD4^+^CD25^+^FoxP3^+^ (**B**) cells and Gr-1^+^CD11b^+^CD11c^−^ (**C**) cells in both non-treated and experimental mice. The percentage of tumor-infiltrating FoxP3^+^CD4^+^CD25^+^ (**D**), IL-10^+^CD4^+^CD25^+^FoxP3^+^ (**E**) cells, Gr-1^+^CD11b^+^CD11c^−^ (**F**), NF-κB^+^CD11b^+^CD11c^−^Gr-1^+^ (**G**), iNOS^+^CD11b^+^CD11c^−^Gr-1^+^ (**H**) cells and number of CD11c^+^ (**I**) and iNOS^+^CD11c^+^ (**J**) were determined using flow cytometry. The presented data represent the mean ± S.E.M. of six mice per group and are representative of three separate experiments. Statistical significance was determined using a Mann–Whitney rank–sum test for graph (**A**), and a paired Student’s *t*-test for graphs (**B**,**C**,**E**–**G**).

We further analyzed immunosuppressive cells in spleens and tumors of metformin-treated and control mice. Data revealed that metformin therapy significantly reduced the percentage of FoxP3^+^ (*p* = 0.008) CD4^+^CD25^+^ cells and IL-10-producing (*p* = 0.001) splenic CD4^+^CD25^+^FoxP3^+^ cells (Figure 5A,B). Moreover, metformin-treated mice exhibited a decrease in the percentage of Gr-1^+^ CD11b^+^CD11c^−^ cells in the spleen (*p* = 0.0001; Figure 5C).

Analyses of the cellular make up of tumor-infiltrating leukocytes detected a reduced percentage of FoxP3^+^ (*p* = 0.001) CD4^+^CD25^+^ cells and IL-10^+^ (*p* = 0.0001) CD4^+^CD25^+^FoxP3^+^ cells in metformin-treated mice compared to the non-treated group (Figure 5D,E). Furthermore, treatment with metformin significantly decreased the percentage of Gr-1^+^ (*p* = 0.0001) CD11b^+^CD11c^−^ cells, nuclear factor kappa B (NF-κB)^+^ (*p* = 0.031) CD11b^+^CD11c^−^Gr-1^+^ cells, and inducible nitric oxide synthase (iNOS)^+^ (*p* = 0.004) CD11b^+^CD11c^−^Gr-1^+^ cells in comparison to the control group (Figure 5F–H). Lastly, metformin notably increased the quantity of CD11c^+^ cells within the tumor environment (*p* = 0.002); however, it decreased the proportion of iNOS^+^ (*p* = 0.002) CD11c^+^ cells isolated from the tumor (Figure 5I,J).

### 2.6. Metformin Administration Demonstrates a Significant Impact on the Expression Profiles of Transcriptional Factors and Chemokines within Splenocytes

**Figure 6:**
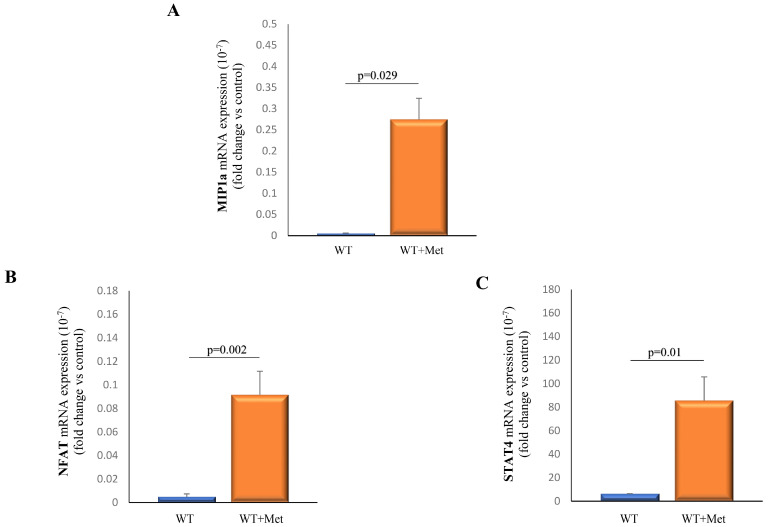
Metformin application significantly increases mRNA expression of MIP1a, NFAT, and STAT4 in splenocytes. The graphs display changes in mRNA levels of MIP1a (**A**), NFAT (**B**), and STAT4 (**C**) in splenic cells from both the control and experimental groups of mice following metformin application. The presented data represent the mean ± S.E.M. of six mice per group and are representative of three separate experiments. Statistical significance for graphs (**A**–**C**) was determined using the Mann–Whitney U test.

Measurement of mRNA expression of macrophage inflammatory protein 1 alpha (MIP1a), signal transducer and activator of transcription 4 (STAT4), and nuclear factor of activated T-cells (NFAT) obtained from splenocytes from metformin-treated and control mice was performed. Our findings revealed a significant upregulation in the expression levels of MIP1a (*p* = 0.029), STAT4 (*p* = 0.002), and NFAT (*p* = 0.01) in splenocytes isolated from metformin-treated mice in comparison to the untreated group (Figure 6A–C).

## 3. Discussion

Our study explores how metformin, a drug known for treating diabetes, can also boost the immune system’s fight against breast cancer by focusing on NKT cells. Through rigorous experimentation, we unveiled compelling evidence of metformin’s capacity to enhance the antitumor functions of NKT cells, delineating a promising path for augmenting breast cancer therapy. Key findings reveal that metformin not only delays the onset of palpable tumors but also attenuates their progression, primarily by modulating the immunosuppressive and cytotoxic landscape within the tumor microenvironment. Highlighting the synergistic impact of metformin on NKT cell activation against breast cancer cells, our study contributes to a nuanced understanding of the drug’s dual role in metabolic and immune regulation.

### 3.1. Metformin’s Multifaceted Role in Cancer Treatment

Extracted from Galega Officinalis, a plant known historically for its antidiabetic properties in medieval European pharmacopeia, metformin has established itself as a primary therapeutic agent for type II diabetes over the past several decades [10]. Recent scientific inquiries have extended beyond its glycemic control capabilities to its impact on oncological outcomes [11]. Studies have documented its efficacy in curtailing the progression and proliferation of a spectrum of cancers, including those affecting the endometrium, breast, colorectal regions, and melanoma [12,13,14].

Since its initial association with reduced cancer incidence in diabetic patients reported in 2005, metformin has been the subject of extensive research exploring its utility as both a preventive measure and an adjunctive therapy in oncology [11]. This body of work elucidates metformin’s multifaceted mechanisms of action, highlighting its influence on cancer cell metabolism, angiogenesis, apoptosis, autophagy, immune modulation, epigenetic alterations, inflammatory processes, and its interaction with the microbiome. Moreover, evidence supports its synergistic potential when combined with conventional cancer treatments, such as chemotherapy, radiotherapy, immunotherapy, and targeted therapies. An accumulating corpus of clinical data not only reaffirms its preventive benefits but also suggests its expanded utility across various stages of cancer management, including neoadjuvant, adjuvant, maintenance, and salvage therapy settings [12,13,14].

Our primary objective was to ascertain whether metformin could, in line with existing research, both delay the emergence and mitigate the advancement of primary breast cancer. Initial observations indicated a markedly faster onset of primary breast tumors in the control cohort of mice relative to those treated with metformin (Figure 1A). Furthermore, the experimental group exhibited a significantly constrained tumor growth compared to their untreated counterparts (Figure 1B). The relevance of these outcomes was validated through the measurement of tumor diameters and the calculation of tumor volumes (Figure 1B,C), underscoring the therapeutic potential of metformin in the context of breast cancer.

### 3.2. Metformin and Immune Modulation

The immune system plays a pivotal role in both the prevention and control of cancer. Research has elucidated the profound impact of metformin on immunological responses, markedly altering the body’s defense mechanisms to suppress tumors through both direct and indirect interactions [15,16]. A notable mechanism through which metformin exerts its anticancer effects is by enhancing the functionality of CD8+ T lymphocytes and mitigating their exhaustion. These cells are crucial for cellular immunity against tumors, capable of expanding and differentiating into effector cytotoxic T lymphocytes (CTLs) that specifically target cancer cells [17,18].

### 3.3. Metformin’s Role in Amplifying NK Cell Response to Cancer

The nuanced relationship between metformin and the innate immune system’s efficiency against cancer cells gains particular relevance in the context of poorly immunogenic tumors, such as those derived from the 4T1 cell line [19,20]. Recent findings from our laboratory have shed light on how metformin significantly bolsters the cytotoxic capabilities of NK cells, highlighting a notable increase in the expression of NKp46^+^, FasL^+^, and IFN-γ^+^ NK cells, alongside a reduction in IL-10-producing NK cells [21]. This indicates metformin’s capacity to enhance NK cell-mediated tumor suppression beyond simple inhibition of immune checkpoints or the indoleamine 2,3-dioxygenase (IDO) pathway. In particular, metformin’s administration was found to markedly upregulate the expression of immunostimulatory microRNAs, miRNA-150 and miRNA-155, while concurrently diminishing the expression of the immunosuppressive miRNA-146a and underscoring a multifaceted mechanism of immune modulation [21].

Delving deeper into the molecular underpinnings of metformin’s impact, studies have illuminated its role in improving NK cell surveillance against cancer. Notably, direct exposure of NK cells to metformin independently augments their cytolytic activity, promoting increased infiltration into tumor sites, a process not contingent on AMP-activated protein kinase (AMPK) signaling [22]. Further, metformin has been shown to induce activation of the Janus kinase 1/2/3 (JAK1/2/3)/signal transducer and activator of transcription 5 (STAT5) and protein kinase B (AKT)/mammalian target of rapamycin (mTOR) pathways in a p38 mitogen-activated protein kinase (MAPK)-dependent manner, diverging from the traditionally acknowledged AMPK-dependent pathways [22]. This was paralleled by enhanced NK cell cytotoxicity against human cervical cancer cells, achieved through the modulation of tumor cell surface expression of NK-cell activating ligands via the phosphoinositide 3-kinase (PI3K)/AKT signaling pathway. Such modulation significantly bolsters NK cell activation, culminating in a potentiated immunological assault on tumor cells [23]. These insights not only emphasize the pivotal role of metformin in augmenting the innate immune system’s capacity to thwart tumor progression but also highlight its potential utility as a synergistic agent in cancer therapy, leveraging the innate immune system to combat tumor growth effectively.

### 3.4. NKT Cells: Orchestrators of Immune Equilibrium in the Tumor Microenvironment

NKT cells represent a unique subset of CD1d-restricted T cells that serve as a crucial bridge between the innate and adaptive branches of the immune system [7]. These cells exhibit a remarkable capacity to quickly respond to a diverse array of glycolipids and stress-related proteins through mechanisms characteristic of both T cells and NK cells [24]. Given their ability to significantly influence immune responses through the secretion of various cytokines, NKT cells play a pivotal role in the immunological surveillance against tumors [25].

At the nascent stages of tumor development, subsets of NKT cells akin to T helper (Th)1 cells possess the ability to promptly activate tumor-specific T cells and NK cells [26]. This activation facilitates the targeted elimination of tumor cells, underscoring the critical role of NKT cells in the early defense against tumorigenesis [27]. However, as tumor progression ensues, the continual stimulation of NKT cells can lead to a state of anergy, characterized by the activation-induced cell death of a fraction of the NKT cell population [28]. This phenomenon not only results in the depletion of a segment of these immune cells but also contributes to the diminished responsiveness of the remaining NKT cells.

Further complicating the landscape of immune response in the context of advancing tumors, the residual population of NKT cells may undergo a functional shift towards subsets that exhibit immunosuppressive characteristics, akin to those observed in Th2 or T regulatory (Treg) cells [28]. Such a transition in the functional orientation of NKT cells contributes to the facilitation of tumor progression and the evasion of immune surveillance by cancer cells.

### 3.5. Metformin and NKT Cells: Bridging Innate and Adaptive Immunity in Cancer Therapy

Recent studies shed light on NKT-mediated tumor inhibition. In animal models of hepatic tumors, Ma et al. showed an increased accumulation of NKT cells and suppression of tumor growth [29]. Besides the possibility of eliminating tumor cells directly, NKT cells have a pivotal role in immunoregulation as they can stimulate or inhibit the immune response [27]. According to these findings, as a continuation of our research, the next goal was to investigate the metformin effect on NKT cells, as a potential antitumor weapon and functional bridge between innate and acquired immunity.

Our research delineates the profound impact of metformin on modulating the immune landscape, particularly within the milieu of NKT cells. Our study identified NKT cells using CD49b/CD3 co-staining, a method supported by numerous immunological studies for effectively distinguishing these cells within the immune landscape [30,31,32]. This approach was chosen based on its established reliability in identifying a broad spectrum of NKT cell populations, particularly within tumor environments where cell phenotypes may differ from those seen in controlled, non-pathological settings. While CD49b and CD3 are recognized markers for NKT cells, it is important to note that our results predominantly reflect the activity and characteristics of Type II NKT cells [33]. Metformin administration was observed to significantly diminish the expression of immunosuppressive markers, including PD-1, FoxP3, and IL-10, in splenic NKT cells, while concurrently augmenting the expression of cytotoxic markers such as CD107a (Figure 3A–C,E). Given that CD107a indicates the cytotoxic activity of NKT cells, its increased expression under metformin treatment suggests enhanced direct cellular cytotoxicity against tumor cells [34]. This dual modulatory effect was mirrored in tumor-infiltrating NKT cells, where a decrease in the percentage of PD-1^+^, FoxP3^+^, IL-10^+^, and KLRG1 MFI^+^ expression was accompanied by an upregulated expression of the activating receptors NKp46 and FasL (Figure 3F–K). Increased percentage of NKp46+ NKT cells and enhanced expression of FasL on NKT cells indicate enhanced cytotoxic capacity of NKT cells resulting in better regulation and elimination of tumor cells [35,36]. Besides T cells and NK cells as primary IFN-γ sources, NKT cells also contribute significantly to the production of this cytokine [37]. Moreover, metformin’s influence extends to the cytokine milieu, with a notable increase in IFN-γ secretion (Figure 3D), a cytokine pivotal for its antitumor properties. This is in alignment with studies highlighting the role of NKT-cell derived IFN-γ in stimulating other immune cells towards tumor eradication [38]. Notably, the interaction between metformin and anti-PD-1 antibody treatment has been shown to induce a surge in IFN-γ production in CD8+ T cells, further substantiating metformin’s role in enhancing the immune response against tumors [39]. Regarding the percentage of PD-1^+^, FoxP3^+^, and IL-10^+^ NKT cells, along with the expression of KLRG1 measured by MFI, it is important to note that FoxP3 and IL-10 are involved in promoting immune tolerance [40,41], PD-1 is a marker of cellular exhaustion [42], and that KLRG1 is linked to immune cell inhibition [43]. Therefore, metformin treatment could potentially enhance NKT cell cytotoxicity against tumors by reducing their immunosuppressive activity. These findings implicate that metformin enhances the tumoricidal phenotype of NKT cells.

Additionally, our findings align with existing research on the elevated expression of CD107a in NK cells treated with metformin [22]. This synergistic enhancement of the immune function by metformin was further evidenced in human studies on tumor-infiltrating lymphocytes (TILs), where a reduction in CD8^+^ effector T cells and FoxP3^+^ T regulatory cells was observed in head and neck squamous cell carcinoma [44], mirroring our observations. Consistent with our previous findings [21], metformin treatment led to a decreased percentage of IL-10-producing NK cells, while significantly elevating the percentage of FasL^+^, NKp46^+^, and IFN-γ^+^ cells. Collectively, these insights underscore the potential of metformin as an adjunct in cancer therapy, leveraging the body’s innate immune mechanisms to combat tumor growth effectively.

### 3.6. Metformin’s Modulation of T Cell Phenotypes: Enhancing the Adaptive Immune Response against Cancer

In addition to NK cells, which are pivotal for the innate immune system’s response against tumors, T cells play an essential role within the adaptive immune system in combating cancerous cells. Reflecting on the multifaceted abilities of NKT cells to both directly and indirectly target and eliminate tumor cells, it becomes evident that NKT cells serve as a critical link between innate and adaptive immunity, stimulating a broad spectrum of immune responses [45]. This basic understanding encourages us to further study how metformin might affect T cell behavior, considering if its impact is direct or happens through its interactions with NKT cells.

Our research aimed to explore how metformin affects the characteristics of splenic and tumor-infiltrating T cells. We found that metformin treatment leads to an increase in the expression of FasL, an inducer of apoptosis, while reducing the levels of immunosuppressive markers such as PD-1 and KLRG1 in splenic T cells (Figure 4A–C). This pattern was also observed in the tumor microenvironment, where there was a noticeable rise in T cells expressing NKp46, FasL, IL-17, and STAT3, along with a decrease in PD-1^+^ and FoxP3^+^ T cells (Figure 4D–J). These results support the idea that metformin enhances the cancer-fighting abilities of T cells, highlighting its potential to influence the adaptive immune response in combating tumors [46].

### 3.7. Metformin’s Immunomodulatory Effects: Diminishing Immunosuppression and Enhancing Tumor Immunity

Further, we meticulously examined the influence of metformin on the systemic balance between pro-inflammatory and anti-inflammatory cytokines, uncovering significant insights into its modulatory effects on the immune environment. Our investigation highlighted a marked decrease in IL-10 levels in metformin-treated mice, pointing towards a lessened immunosuppressive environment, while levels of TNFα, TGF-β, IL-2, IL-4, and IL-13 remained unaffected (Figure 2). This significant decrease in IL-10, typically secreted by Tregs and MDSCs, prompted us to delve deeper into metformin’s effect on these cell populations. Table 1 provides a detailed overview of the specific immune cell subsets evaluated in this study, including CD11b^+^CD11c^−^Gr-1^+^ MDSCs, CD4^+^CD25^+^FoxP3^+^ Tregs, CD3^+^CD49^−^ T cells, and CD3^+^CD49^+^ NKT cells.

Our results showed a reduction in the population of Gr-1^+^CD11b^+^CD11c^−^ MDSCs, and a decrease in the percentage of FoxP3^+^CD4^+^CD25^+^ Tregs, and IL-10^+^ in CD4^+^CD25^+^FoxP3^+^ Tregs within the spleen (Figure 5A–C). Furthermore, tumor-infiltrating leukocyte analysis revealed a lower percentage of FoxP3^+^ and IL-10^+^ Tregs as well as a decreased percentage of Gr-1^+^CD11b^+^CD11c^−^, NFκB^+^, and iNOS^+^CD11b^+^CD11c^−^Gr-1^+^ MDSCs in metformin-treated mice (Figure 5D–H), aligning with Qin et al.’s findings that metformin treatment via NF-κB inhibition suppressed MDSC migration [47].

Moreover, another study highlighted the role of NKT cells, through interactions with DCs, in reducing MDSC numbers and suppressing their immunosuppressive phenotype [48], suggesting that metformin’s anticancer mechanism may leverage the NKT cell and dendritic cell axis. NKT cells exert considerable influence over T cell responses, mainly through the secretion of a broad array of cytokines and chemokines and by providing co-stimulatory signals that enhance T cell activation and proliferation.

Activation of specific NKT cell subsets with α-GalCer has been shown to lead to full dendritic cell maturation, significantly amplifying CD4^+^ and CD8^+^ T cell activities [49]. Our analysis not only demonstrated an increased percentage of dendritic cells but, importantly, a reduced proportion of immunosuppressive iNOS-producing dendritic cells within the tumor environment of metformin-treated mice (Figure 5I,J). These findings imply that metformin’s modulatory effects on the immune system may involve intricate interactions between NKT cells and dendritic cells, thus potentially altering T cell phenotypes to bolster anticancer immunity [50].

### 3.8. Metformin’s Impact on NKT Cell Activation Markers

In the final phase of our study, we focused on assessing the expression levels of crucial molecules instrumental in the activation and function of NKT cells. Specifically, we investigated STAT4, recognized for its necessity in NKT cell response to IL-12 and differentiation into Th1 and Th17 phenotypes, serving as a key indicator of NKT cell activation [51]. Alongside STAT4, NFAT plays a vital role in the activation of NKT cells, marking it as a significant transcription factor in this context [52]. Additionally, we evaluated MIP1a, a chemokine produced by NKT cells, essential for recruiting various immune cells to sites requiring immune intervention [53].

**Figure 7:**
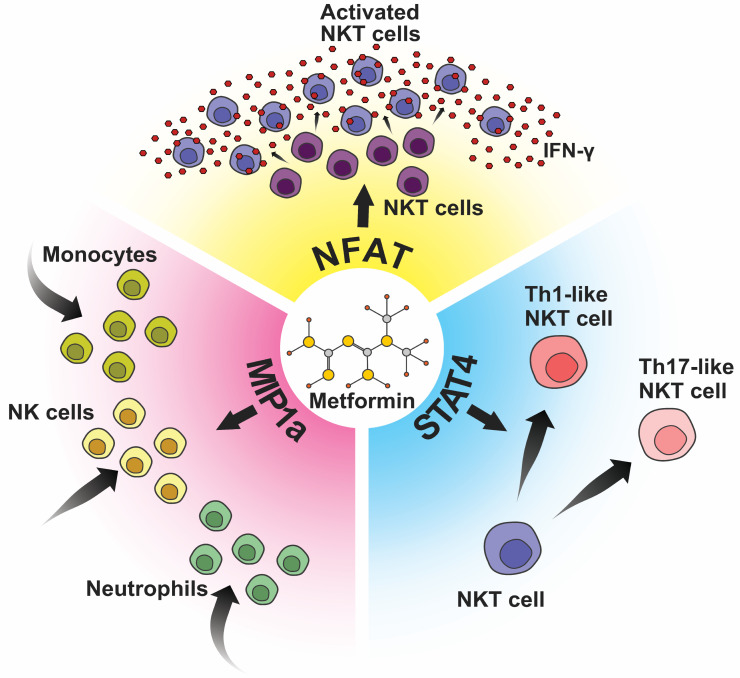
Schematic illustration of proposed metformin’s effect on NKT cells. Metformin increases the expression of: MIP1a (pink), which leads to increased recruitment of immune cells, mostly NK cells, monocytes, and neutrophils; STAT4 (blue), which potentiates differentiation of NKT cells into Th1-like NKT cells and Th17-like NKT cells; and NFAT (yellow) which leads to increased NKT cell activation and increased production of IFN-γ.

Through polymerase chain reaction (PCR) testing, we observed a marked upregulation in the expression levels of STAT4, NFAT, and MIP1a within metformin-treated splenocytes. As these molecules are crucial in NKT cell signaling pathways, these results may suggest that metformin treatment potentially primes NKT cells for heightened activation and engagement in immune responses against tumors (Figure 6A–C). These observations were further illustrated in a schematic representation (Figure 7), providing a visual summary of metformin’s potential influence on NKT cell activation and its consequent impact on the broader immune system dynamics in combating cancer.

## 4. Materials and Methods

### 4.1. Experimental Design, Tumor Induction, and Metformin Administration

All experiments were carried out on female BALB/C wild-type (WT) mice. Mice (6 to 8 weeks old) were raised in the Faculty of Medical Sciences animal facility in a standard laboratory setting (12 h light/12 h dark cycle, 22 ± 2 °C temperature, and a 51 ± 5% relative humidity. Before and throughout the whole experiment, the animals were allowed free access to ordinary laboratory food and water. Before the experiment began, mice were randomly separated into one of two groups:

(1) wild-type BALB/C mice with induced mammary tumors which were given 200 mg/kg of metformin diluted in 100 μL of distilled water daily;
(2) wild-type BALB/C mice which were induced mammary tumors and given 100 μL of distilled water daily.

Tumors were induced in both groups by using weakly immunogenic murine mammary carcinoma-4T1 cells which are syngenic for BALB/C mice. Cells were bought from the American Type Culture Collection (ATCC) and cultured in Dulbecco’s Modified Eagle’s Medium (DMEM) enriched with 10% heat-inactivated fetal bovine serum (FBS), L-glutamine (2 mmol/L), penicillin-streptomycin (1 mmol/L), and mixed nonessential amino acids (1 mmol/L, Sigma). Cultured 4T1 cells were isolated via treatment with trypsin (0.25%) and ethylene diamine tetra-acetic acid (EDTA, 0.02%) in phosphate-buffered saline (PBS, PAA Laboratories GmbH, Etobicoke, Canada) and washed twice before they were used in all experiments, as originally described in a study by Jovanovic et al. [20]. Trypan blue exclusion was used for the estimation of the viability of the 4T1 cells. In all in vitro and in vivo experiments, we exclusively used cell suspensions that exhibited a viability rate greater than 95%. Tumors were initiated by administering 5 × 10^4^ 4T1 cells diluted in 50 μL of complete medium into each mouse’s fourth mammary fat pad (MFP).

Metformin was given to mice on a daily basis, commencing on the first day of the experiment, following the administration of 4T1 tumor cells. Each mouse received a dose of 200 mg/kg diluted in 100 μL, intraperitoneally. The experiment lasted 35 days.

The dataset includes results from six mice in each experimental group, collected throughout three separate experiments.

The Animal Ethics Committee of the Faculty of Medical Sciences, University of Kragujevac, Serbia, evaluated and approved all studies. The study was performed in accordance with the ARRIVE principles and the EU Directive 2010/63/EU on animal research.

### 4.2. Palpable Tumor Appearance and Growth

In order to assess the palpable tumor appearance and progression, tumors were monitored every day, by palpation, and the tumor diameter was measured using an electronic caliper specifically on days 12, 15, 19, 22, 28, and 35. On the 35th day of the experiment, the mice were euthanized. Primary tumors alongside with spleens were surgically removed. The tumor volume (V) was calculated using the formula V = LW2/2 where L represents the length of the tumor and W represents the width. Furthermore, we drew a sample of blood from the abdominal aorta.

### 4.3. Flow Cytometric Analyses of Splenocytes and Tumor-Infiltrating Leukocytes

Initially, on the 15th day of the experiment, mice were euthanized, and TILs and splenocytes were extracted from both metformin-treated and untreated animals. Flow cytometry assays were used to evaluate the samples. While the single-cell suspensions from primary tumors were isolated via enzymatic digestion, samples from the spleen were isolated using mechanical dispersion through cell strainers (BD, Pharmingen, USA). Cells were exposed to fluorochrome-labeled anti-mouse antibodies specific for CD3 (PerCP-Cy5.5), CD4 (PerCP-Cy5.5), CD11b (FITC/PE), CD11c (APC), CD25 (FITC), CD45 (APC), CD49b (FITC), Foxp3 (PE/APC), Gr-1 (PerCP-Cy5.5), PD-1 (APC), KLRG1 (PE), FasL (PE), NKp46 (APC) or the corresponding isotype controls (BD Pharmingen, NJ/Invitrogen, Carlsbad, CA). Intracellular markers were stained with TNF-α (PerCP-Cy5.5), CD107a (FITC), perforin (APC), Foxp3 (PE/APC), IFN-γ (PE), granzyme (PE), IL-10 (PE/APC), IL-17 (PerCP-Cy5.5), STAT-3 (PE), and iNOS (PE) (BD Pharmingen/BioLegend/eBiosciences). In order to identify the NFκB transcription factor, we used a Rabbit Anti-NF-kB p65 antibody obtained from Abcam. Subsequently, we applied a secondary FITC-conjugated DNK anti-rabbit IgG monoclonal antibody. Samples were prepared by stimulating the cells with phorbol 12-myristate 13-acetate (PMA; 50 ng/mL; Sigma-Aldrich, St. Louis, MO, USA), Ionomycin (500 ng/mL; Sigma-Aldrich), and GolgiStop (BD Pharmingen, San Diego, CA, USA) for 4 h at 37 °C, 5% CO_2_. Flow cytometry was performed using a FACSCalibur Flow Cytometer (BD Biosciences, San Jose, CA, USA), and the expression levels of complement receptors and regulators were analyzed using FlowJo v10.7.2. (Tree Star, Ashland, OR, USA).

### 4.4. Serum Level of Cytokines

The serum concentration of cytokines in the mice was determined by measuring levels of TNFα, TGF-β, IL-2, IL-4, IL-10, and IL-13. This was accomplished using enzyme-linked immunosorbent assay (ELISA) kits that were highly sensitive and specific for the mouse cytokines, as provided by R&D Systems. The manufacturer’s instructions were followed meticulously during the assay.

### 4.5. Quantitative Real-Time Polymerase Chain Reaction (RT-qPCR)

The initial step was to isolate total RNA from splenocytes using the Trizol reagent (TRI Reagent^®^ Solution, Applied Biosystems, Foster City, CA, USA). We generated cDNA using a reverse transcriptase (a High-Capacity cDNA Reverse Transcription Kit (Applied Biosystems, Foster City, CA, USA), in accordance with the manufacturer protocol. A volume of 1 mL of cDNA was used for the analysis of gene expression, which was conducted via quantitative PCR in real time. The RT-qPCR was performed using a Mastercycler^®^ ep realplex (Eppendorf, Hamburg, Germany) and microtiter plates with 96 wells (Twin. tec. real-time PCR plates 96, Eppendorf). The expression of STAT4 and NFAT transcriptional factors and MIP1a chemokine was analyzed using oligonucleotide primers from Table 2. The mRNA expression of the target genes was normalized to GAPDH expression, and the fold change was calculated as 2^−ΔΔCt^ (cycle threshold).

### 4.6. Statistical Analysis

Statistical analysis was conducted using commercial software, specifically SPSS version 23.0. All comparisons among both control and experimental groups were performed using the Student’s *t*-test, Mann–Whitney U-test, ANOVA, or Kruskal–Wallis test, as appropriate. A log-rank test was used to assess disparities in the appearance of primary tumors using Kaplan–Meier analysis. The data are presented as the mean ± standard error of the mean. Statistically significant differences were defined as a *p*-value of <0.05.

## 5. Conclusions

**Figure 8:**
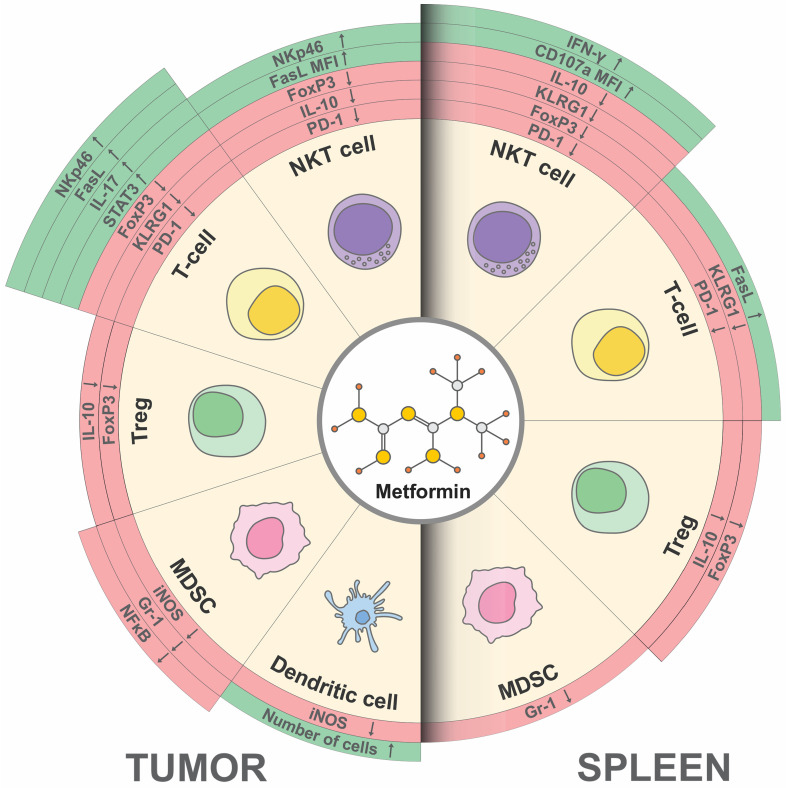
Schematic representation illustrating the metformin’s multifaced impact on the functional phenotype of various immune cell populations. NKT cells and T cells: Metformin potentiates the cytotoxic phenotype of tumor and splenic NKT cells and T cells, suppresses the expression of inhibitory surface receptors, and inhibits the production of immunosuppressive cytokines. Treg Cells: Metformin suppresses the inhibitory phenotype of Tregs. MDSCs: Metformin suppresses the inhibitory phenotype of MDSCs. Dendritic cells: Metformin suppresses the inhibitory phenotype of dendritic cells in the tumor microenvironment. The arrows in the schematic are used to depict changes in the expression of the surface receptors, the percentage of specific cell types, and the production of certain cytokines: arrows pointing downwards indicate a decrease, while arrows pointing upwards indicate an increase. The color coding for these arrows is as follows: red represents a decrease, and green represents an increase.

Collectively, metformin delayed the appearance of palpable tumors and slowed the progression of 4T1 breast cancer. Metformin administration directly altered the phenotype of NKT cells which can further stimulate T cells directly or over dendritic cells as well as inhibit the immunosuppressive effect of MDSCs and Tregs. The possible explanation is that metformin probably directly affects the functional phenotype of NKT cells by changing the expression of signaling molecules and transcription factors STAT4, NFAT, and MIP1a and that NKT cells through reduced production of immunosuppressive IL-10 affect DCs and thus indirectly effector T lymphocytes. Also, metformin probably both directly and indirectly, via NKT cells, slows down the accumulation of immunosuppressive MDSCs and Tregs in the tumor microenvironment. The limitation of our study is the use of PCR testing on splenocytes rather than directly on purified NKT cells. In order to fully confirm the contributions of NKT cells to the observed effects, further experiments should focus on isolated NKT cells. The collective influence of metformin on the functional phenotype of NKT cells, T cells, Tregs, MDSCs, and dendritic cells is graphically summarized in Figure 8. Future studies need to clarify the precise mechanism of NKT cells acting in an antitumor immune response.

## Figures and Tables

**Table 1:** Specific Immune Cell Subsets in Tumor Immune Microenvironment.

Cell Marker Combination	Cell Type Identified	Role in Tumor Development	Reference
CD11b^+^CD11^−^	Myeloid-Derived Suppressor cells (MDSCs)	MDSCs are important in establishing an immunosuppressive tumor microenvironment. They inhibit the cytotoxic functions of T cells and NKT cells and recruit Tregs, which collectively contribute to tumor immune evasion and progression.	[30]
CD4^+^CD25^+^	Regulatory T cells (Tregs)	Tregs maintain immunological tolerance and suppress anti-tumor immune responses by inhibiting effector T cells and NKT cells through the secretion of inhibitory cytokines like IL-10 and TGF-β. Their suppressive activities are associated with a reduction in overall survival rates in various cancers.	[28]
CD3^+^CD49^−^	T cells	T cells are essential for adaptive immunity and can directly attack tumor cells. Their activity can be suppressed by Tregs and MDSCs within the immunosuppressive tumor microenvironment. Enhancing their activation and persistence can be pivotal for effective immunotherapy.	[39]
CD3^+^CD49^+^	Natural Killer T cells (NKT) cells	NKT cells can function both as tumor-promoters or tumor-inhibitors depending on their subtype and the cytokines they are exposed to. They interact dynamically with MDSCs, Tregs, and dendritic cells, playing a complex role in balancing immune surveillance and tumor promotion.	[29]

**Table 2:** Primers used in polymerase chain reaction (PCR).

Gene	Primer	Sequences (5′-3′)
*GAPDH*	Forward	GTCTCCTCTGACTTCAACAGCG
*GAPDH*	Reverse	ACCACCCTGTTGCTGTAGCCAA
*MIP-1a*	Forward	CTCACCTGCTGCTACTCATTC
*MIP-1a*	Reverse	CATGATGTTGAGCAGGTGACAGA
*NFAT*	Forward	GGTGCCTTTTGCGAGCAGTATC
*NFAT*	Reverse	CGTATGGACCAGAATGTGACGG
*STAT4*	Forward	GCTGAATGACGGTGCAAACGG
*STAT4*	Reverse	GACAGTGGGAGTGGCACCTT

## Data Availability

The data presented in this study are available on request from the corresponding author. The data are not publicly available due to the established practice of the authors.
